# Ballpark Estimates of Budget Space for Health Workforce Investments in the 47 Countries of the WHO African Region: A Modelling Study

**DOI:** 10.1177/11786329251320429

**Published:** 2025-02-15

**Authors:** James Avoka Asamani, San Boris Kouadjo Bediakon, Hamza Ismaila, Sunny Okoroafor, Regina Titi-Ofei, Adam Ahmat, Juliet Nabyonga-Orem, Ogochukwu Chukwujekwu, Kasonde Mwinga

**Affiliations:** 1 World Health Organization Regional Office for Africa, Brazzaville, Republic of Congo; 2 Centre for Health Professions Education, North-West University, Potchefstroom, South Africa; 3 The Global Fund to Fight AIDS Tuberculosis and Malaria, Grand-Saconnex, Switzerland

**Keywords:** Fiscal space, budget space, health workforce, prioritisation for health, prioritisation for health workforce, health workforce expenditure envelop, health workforce investment

## Abstract

**Introduction:**

The needs-based requirement for health workers in the 47 countries of the World Health Organization’s African Region is estimated to be 11.8 million by 2030, and the supply will fail to meet the need, leaving an anticipated shortage of 6.1 million by 2030. However, several countries are also having a situation whereby trained health workers cannot be employed due to budget space constraints. This paper sought to explore the level of prioritisation of health and health workforce in government spending, estimate the budget space potential for investing in the employment of health workers using scenario analysis and estimate the budget space gap if all the trained health workers were to be employed.

**Method:**

Building on previous work using publicly available data, the study modelled 3 scenarios of health workforce investment (expenditure): (1) business as usual scenario in which it was assumed the level of prioritisation for health from the overall envelope for government spending on consumption, and the prioritisation of HWF from the health sector allocation/budget will be constant. Thus, expansion in the budget space will be a function of economic growth, (2) HWF prioritisation scenario, in which it was assumed that all parameters were held constant, but countries will prioritise at least 43% of their health budget for HWF employment – in line with the regional average and (3) health prioritisation scenario in which it is assumed that countries will prioritise at least 15% of public sector consumption (general government spending) for health – in line with the Abuja target – but maintain prevailing levels of HWF prioritisation from the health budget. A 3-step model was developed first to estimate the annual general government consumption expenditure envelope, from which the public expenditure envelope for health was estimated and then the potential expenditure envelope for the health workforce.

**Results:**

On health workforce budget space, the ‘business as usual’ scenario, showed an estimated expenditure envelope for HWF from all sources could increase from $20.85 billion in 2022 to $31.81 billion by 2030, driven by macroeconomic factors like GDP growth. However, this could be affected by uncertainty in overseas development assistance. In ‘HWF prioritisation’ scenario, prioritising at least 43% of the health budget for health workforce (HWF) employment increased the HWF envelope by 28%. In the ‘health prioritisation’ scenario, prioritising at least 15% of public sector consumption expenditure for health (but maintaining the prevailing levels of prioritisation for HWF) could yield $55.32 billion for health workforce employment by 2030. In 2022, there was a 43% deficit in the current spending level to employ and pay the remuneration of all trained health workers in the Region, taking into account government and private sector spending as well as overseas development assistance. This financing gap translates into unemployed health workers, estimated to be 27% (95% CI: 14%-39%) based on data from a subset of 10 countries conducting health labour market analyses.

**Conclusion:**

Better prioritising health within the overall government expenditure coupled with highly prioritising the health workforce to at least regional average levels of 43% within the health budget could make a difference in the investments needed to tackle the health workforce challenges. This should, however, be underpinned by ensuring efficient and accountable use of the resources.

## Introduction

The 47 countries in the World Health Organization’s (WHO) African Region have made modest progress in health workforce development over the last 2 decades, alongside improvements in service coverage. There has been increased investment in training and education infrastructure, increasing the number of health professions education institutions to about 4000 from less than 1000 in 2005. This culminated in an increase in the stock of health workers from 1.6 million in 2013 to about 4.3 million in 2018 and 5.1 million by 2022.^1^ Nevertheless, the 47 countries in the WHO African Region continues to grapple with several challenges associated with health labour market failures or mismatches that require urgent investment actions.

Recent studies estimate that the Region will likely face a 6.1 million needs-based shortage of health workers by 2030, of which 5.3 million will be for Doctors, Nurses, Midwives, Pharmacists and Dentists.^2,3^ The WHO African Region is estimated to have the largest share (52%) of the global workforce shortages by 2030.^2^ In 2018, the Region’s density of 29.3 health workers across all occupations (including community health workers) per 10 000 population was considered insufficient to attain even 70% of the UHC index targets by 2030, which at the time required at least 134 per 10 000 population.^4^

The African Region has recorded a remarkable expansion by 70% in its training output for health professionals, increasing from 150 000 graduates in 2018 to over 255 000 in 2022. However, nearly 1 in 3 (about 27%) of the health workers are estimated to be unemployed or under-employed in precarious jobs.^5^ The high level of unemployment has been attributed to an estimated 43% shortfall in the public sector budget space required for adequate employment of health workers being trained.^6^ Also, despite contributing to over 40% of the training of health workers in the Region, the private sector is recruiting approximately only 22%.^7^ Similarly, overseas development assistance has plateaued since 2011, and the bulk, 55%, is channelled to in-service training and ad hoc health worker incentives rather than strategic investment in health workforce education and creating new employment for the trained health workers.^8^

Against a backdrop of inadequate spending on health by countries, the main underlying challenge has been a limited or inadequate capacity to invest in the health workforce in some countries, while in others, there is a need for more prioritisation of health workforce investments within the existing budgets of ministries of health. Although there are elaborate strategies to address some of the challenges, these plans often need to be funded. For example, an analysis of human resources for health strategic plans in the Region found that 74% of countries have costed plans in place, but 41% still need to make a financial commitment to dedicated funding for the implementation. Consequently, the average implementation rate of HWF strategic plans is often between 35% and 40% in the Region.^9^ The aforesaid is a clarion call for accelerated advocacy and policy dialogue to enhance sustained prioritisation and investment in the health workforce to address the population’s health needs and challenges.

In 2022, the WHO Regional Office for Africa (WHO AFRO) conducted a fiscal space modelling for the health workforce in the East and Southern Africa sub-region, which demonstrated sluggish budget space prospects for the health workforce, suggesting that absorbing the current supply of health workers required up to 43% increase in public spending of health workers,^6^ leaving several countries facing a situation where trained health workers cannot be employed due to budget space constraints.^10-13^ However, pre-pandemic data was used for the analysis, which needs to be updated to provide insights into the impact of the COVID-19 pandemic on health workforce investments in the wake of debt crises and austerity measures taken by governments to contain declined economic and fiscal prospects.

Estimating government fiscal and budget space for health (and the health workforce) is complex, especially during economic uncertainty.^6,14-16^ As a result, several fiscal space analyses have hesitated to estimate the anticipated volume of potential health workforce expenditure envelop. Complexities notwithstanding, even imprecise ballpark estimates (order of magnitude estimates) have stimulated dialogue on health workforce investments.^6^

This paper sought to (a) explore the level of prioritisation of health and health workforce in government spending, (b) estimate the budget space potential for investing in the employment of health workers using scenario analysis and (c) estimate the budget space gap if all the trained health workers were to be employed.

## Methodology

### Estimating the resource envelope for investing in the health workforce

**Figure 1.**
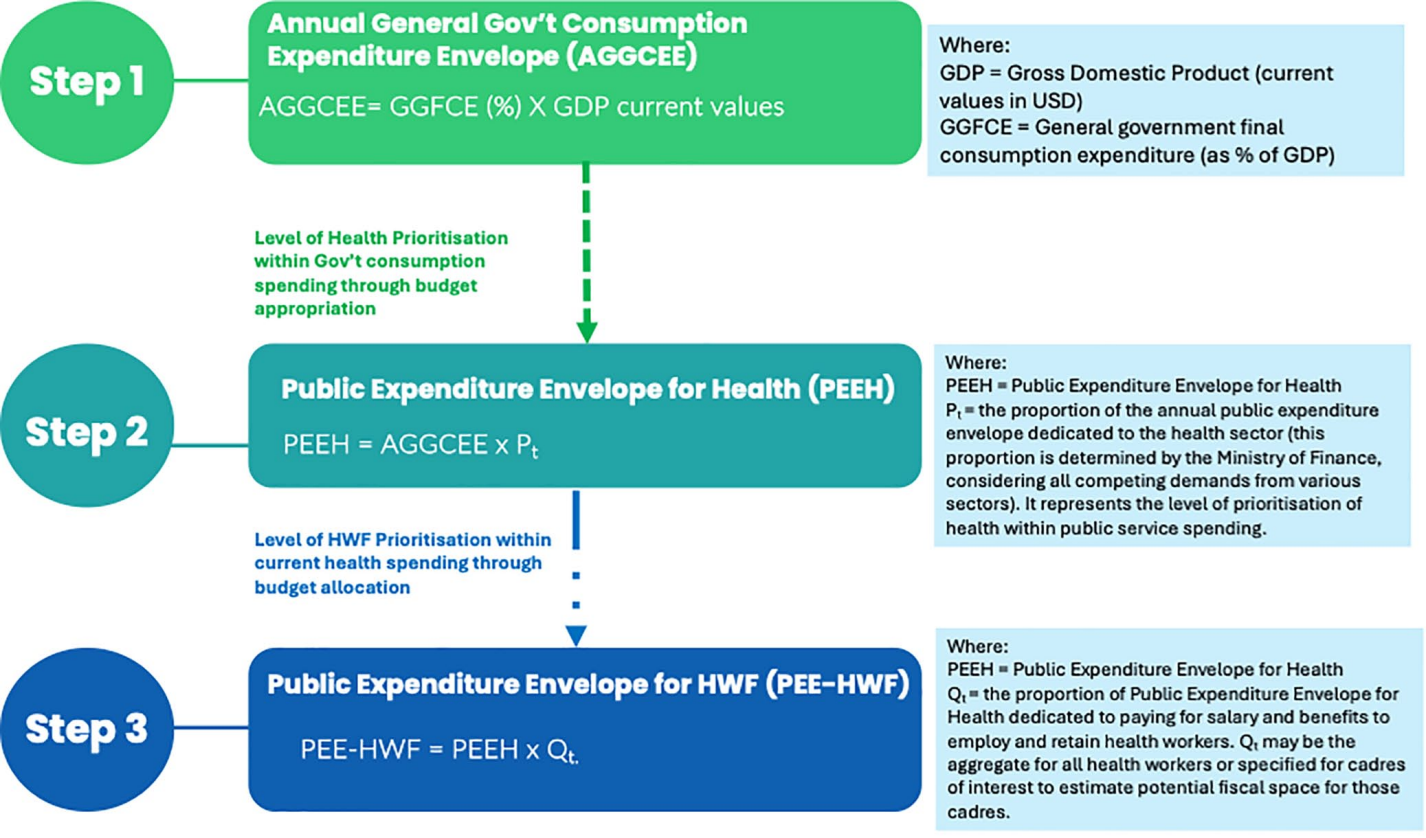
Simplistic 3-step model guiding ballpark estimates of the expenditure envelope for the health workforce. *Source.* Adaptation from Barroy and Gupta^14^ and Ismaila et al.^19^

Building on recent conceptual discussions on budget space analysis^14,16,17^ and empirical applications for the health workforce,^6,18^ we adopted a 3-step model to make guided ballpark estimates of the expenditure envelope for the health workforce (see Figure 1).

#### Step 1: estimating the overall government fiscal space

According to the International Monetary Fund (IMF)^20^ and other authorities on public finance, the overall fiscal room for government spending is influenced by several factors, such as the size of the country’s GDP, the rate of growth of the economy, the volume of public debt, domestic revenue generation capacity and the ability to borrow domestically or internationally.^15,21^ Barroy and Gupta^14^ proposed a process for the estimation of fiscal room for government spending, which we contextualised as a function of (a) Gross Domestic Product, (b) tax to GDP ratio, (c) level of borrowing (d) Debt service or re-payments of principals and interests and (e) Statutory Payments. Equivalently, the general government’s final consumption expenditure (GGFCE) as a percentage of GDP can be used to proxy the overall envelope for government spending on consumption, including in the social sectors such as health. The World Bank defines the GGFCE (formerly general government consumption) to include all government current expenditures for purchases of goods and services (including compensation of employees) as well as most expenditures on national defence and security but excludes government military expenditures, which are classified as part of government capital formation.^22^

(1) $$\begin{array}{l} Annual\;General\;Gov,t\;Consumption\;Expenditure\;\\ Envelope\;(AGGCEE)=(GDP\times GGFCE\;as\;\text{\%}\;of\;GDP) \end{array}$$

#### Step 2: Estimating the public expenditure envelope for health (PEEH)/budget space for health

A prioritised portion of a government’s overall consumption expenditure envelope is usually allocated for current health expenditures. The proportion of the public expenditure prioritised for health is often a result of a complex political economy of budget negotiations between the Ministry of Finance and the Ministry of Health and decisions made by the legislature during legislative appropriation of the public sector budget. This is illustrated in equation (2), where PEEH represents the Public Expenditure Envelope for Health, while P_t_ is the proportion of the annual public expenditure envelope dedicated to the health sector. In essence, the Public Expenditure Envelope for Health (PEEH) reflects budgetary allocation to health and if budget execution and fidelity is high, it would be coterminous with the General Government Health Expenditure (GGHE).^14^

(2) $$PEEH=AGGCEE\times P_{t}$$

Authors have discussed how the amount of government funds allocated to healthcare reflects the focus on health and its importance in public service spending.^6^ While there is no universal agreement on the specific percentage of public spending that should be allocated to health, African governments in 2001 committed to allocating 15% of general government expenditure (popularly known as the Abuja declaration).^23,24^

#### Step 3: Estimating the expenditure envelope for the health workforce

The public expenditure envelope for health – often termed as General Government Health Expenditure (GGHE) covers various aspects of the public health sector, such as the health workforce, infrastructure, medicines, technology, information systems, management and service delivery. The allocation for the health workforce and other areas is influenced by the GGHE size and the policy priority placed on health workforce employment within GGHE (p. 3).^6^ Legislative guidance and restrictions sometimes dictate how much can be directed towards the health workforce. While there is no global standard for the percentage of health budget designated to the health workforce, globally, it’s around 57%,^6^ with Africa averaging about 43%.^6,25^ From the PEE for HWF, the contribution of private sector and overseas development assistance to HWF employment are added based on prevailing patterns of their contribution.^26^

(3) $$PEE\;for\;HWF=PEEH\times Q_{t}$$

Whereby,

- PEEH is the Public Expenditure Envelope for Health (or equivalently, the general government health expenditure (GGHE),
- Q _t_ is the proportion of the public expenditure envelope for health dedicated to paying salaries and benefits to health workers. Q _t_ may be the aggregate for all health workers or specified for occupation(s) of interest.

#### Data sources

The data used to estimate HWF budget space in the 47 countries in the WHO African Region were based on the latest available year for each country and for the particular indicator, and obtained from different databases, reports and peer-reviewed literature (see Table 1).

**Table 1.** Data sources.

#	Variable	Data Source (latest available year for each year)
1	Gross Domestic Product (GDP)	International Monetary Fund^27^
**2**	Annual General Gov’t Consumption Expenditure Envelope (AGGCEE)	World Bank’s World Development Indicators^28^
**3**	General government final consumption expenditure (% of GDP)	Global Health Expenditure Database^29^
**4**	Domestic General Government Health Expenditure (GGHE-D) as a per cent of General Government Expenditure (GGE)	Global Health Expenditure Database^29^
**5**	Percentage of health budget spent on HWF	Toure et al^26^; HLMA Reports; Budgets Acts / National Budgets for fiscal years
6	Private sector contribution to HWF remuneration	Toure et al^26^
7	Contribution of overseas development assistance to HWF remuneration	Toure et al^26^

#### Exploring the level of prioritisation for health workforce spending

We conducted a descriptive analysis of the level of prioritisation of health and health workforce spending by plotting the proportion of General Government Health Expenditure (GGHE) from General Government Expenditure (GGE) against the percent of current health expenditure (CHE) spent on health workforce. This produced 4 distinct categories (quadrants) of countries which interpreted these quadrats as follows:

♦ **Group 1** countries were those higher than the regional average level of prioritisation of health within Government spending but less than the regional average level of prioritisation of HWF within the health spending.
♦ **Group 2** countries were those with higher than the regional average level of prioritisation of health within Government spending and also higher than the regional average level of prioritisation of HWF within the health spending.
♦ **Group 3** countries had lower than the regional average level prioritisation of health within Government spending and also lower than the regional average level of prioritisation of HWF within the health spending.
♦ **Group 4** countries had lower than the regional average level of prioritisation of health within Government spending but higher than the regional average level of prioritisation of HWF within the health spending.

#### Scenarios of budget space for health workforce employment

Three scenarios of budget space were conducted – ‘Business-as-Usual’, ‘HWF Prioritisation’, and ‘Health Prioritisation’.

1. **Business as usual scenario**: In this baseline scenario, we assumed constant level of prioritisation for health from the General Government Final Consumption Expenditure (GGFCE), which we used as a proxy of the overall envelope for government spending on consumption, including the social sectors such as health. Also, the prioritisation of HWF from the health sector allocation/budget was held constant. Thus, the only driver of the budget space in this scenario is macroeconomic factors such as the growth rate of Gross Domestic Product (GDP) as projected by the International Monetary Fund’s World Economic Outlook.

(4) $$\begin{array}{l} PEE\;for\;HWF\\ \;\;\;\;\;\;\;\;\;\;=\left[(GDP\;\times \;GGFCE\;as\;\%\;of\;GDP)\times P_{t}\right]\times Q_{t}\;.\;.\;. \end{array}$$

2. **HWF prioritisation scenario:** In this alternative scenario, all parameters were held constant, but countries were assumed to prioritise at least 43% ( $P_{t}\geq 43\%$ ) of their health budget for HWF employment – in line with the regional average. This scenario is expressed as shown in equation (5).

(5) $$\begin{array}{l} PEE\;for\;HWF\;\\ \;\;\;\;\;\;\;\;\;\;\;\;=\left[\left(GDP\;\times \;GGFCE\;as\;\text{\%}\;of\;GDP\right)\times (P_{t}\geq 43\%)\right]\;\times Q_{t}\;.\;.\;. \end{array}$$

3. **Health Prioritisation Scenario:** In the third scenario, we assumed countries prioritised at least 15% ( $Q_{t}\geq 15\%$ ) of public sector consumption (general government spending) for health – in line with the Abuja target – but maintained prevailing levels of HWF prioritisation from the health budget.

(6) $$\begin{array}{l} PEE\;for\;HWF\;=\left[(GDP\;\times \;GGFCE\;as\;\%\;of\;GDP)\times P_{t}\right]\\ \;\;\;\;\;\;\;\;\;\;\;\;\;\;\;\;\;\;\;\;\;\;\;\;\;\;\;\;\;\;\;\;\;\;\;\;\;\;\;\;\;\times (Q_{t}\geq 15\%)\;.\;.\;. \end{array}$$

#### Estimating the budget space gap to absorb all the trained stock of health workers.

The median salary of health workers was used to multiply with the reported stock of health workers for 2022 and the projected supply for 2030. The projected supply of health workers was taken from a related study.^3^ The funding gap was estimated as the difference between the estimated public expenditure envelop as estimated in equations (4) to (6).

## Results

### Descriptive analysis of variables

The baseline year of the analysis was 2022. The average general government’s final consumption expenditure represents, 14.86% of Gross Domestic Product (GDP) across the 47 countries in the WHO African region, varying from 3.95% to 36.41% (Table 2). Thus, roughly 15% of the annual GDP is spent on purchasing goods and services and paying the remuneration of public sector employees (Table 2). From this, the median General Government Consumption Expenditure Envelop (GGCEE), also known as General Government final consumption expenditure, was US$2.4 billion among the 47 countries, with a wide variation from US$126.2 million to US$10.8 billion.

**Table 2.** Descriptive statistics of variables.

Variable	Observations	Median	Mean	Std. dev.	Minimum	Maximum
General Government Consumption Expenditure Envelop (GGCEE) – current in Million USD; 2022 data)	47	2373.47	7824.10	19 089.49	126.19	10 8455.62
General government’s final consumption expenditure (GGFCE) as of % of gross domestic product (GDP; %) – (2022 data)	47	15.14	14.86	6.83	3.95	36.41
GGHED as % of GGE	47	6.85	7.29	2.92	2.11	15.30
The proportion of health expenditure allocated to remuneration of health workers (Wage bill; %) – latest available year for each country.	47	47.30	43.37	14.77	11.67	71.08
Private sector contribution to spending on health workforce remuneration (%) – latest available year for each country.	33	16.67	22.68	21.09	<1	66.72
Overseas development assistance (ODA) contribution to HWF Remuneration (%) – latest available year for each country.	33	13.24	16.94	16.52	0.18	67.19

In 14 countries where data for indicators were not publicly available, inputation was done using the regional median for 2 variables.

From the GGCEE, countries in the WHO African Region prioritise about 7.3% for health (3.95%-36.41%) – the regional average. Thus, countries prioritise less than the Abuja target of 15% of the overall government consumption expenditure for health. However, a few countries prioritise more than the Abuja target. From health spending, countries prioritise around 43% to pay health workers salaries, allowances, and other benefits. This, however, varies widely from 11.67% to 71.08%.

The private sector plays a role in recruiting health workers in countries, and their contribution is around 22.68% in 33 countries where data was available. In some countries, the private sector is the leading employer with a share above 50% (ie, 66.72%). Also, overseas development agencies (ODA) contribute significantly to the payment of remuneration in some countries, hence shaping the health labour market. About 16.94% of the wage bill of countries is covered by external partners. However, depending on the countries, this share can be as minimal as 0.18% with less ODA dependence or as high as 67.19% for highly dependent countries on development assistance (see Table 2).

### Levels of prioritisation for health and health workforce investments

The latest available data show that, countries in the WHO African region spend about 7% of the general government health expenditure on health, which ranges from 2.1% to 15.3%. Also, the countries prioritise about 43% (range: 12%-71%) of health expenditure for health workforce remuneration and wages, which determines the levels of employment of health workers in the countries.

**Figure 2.**
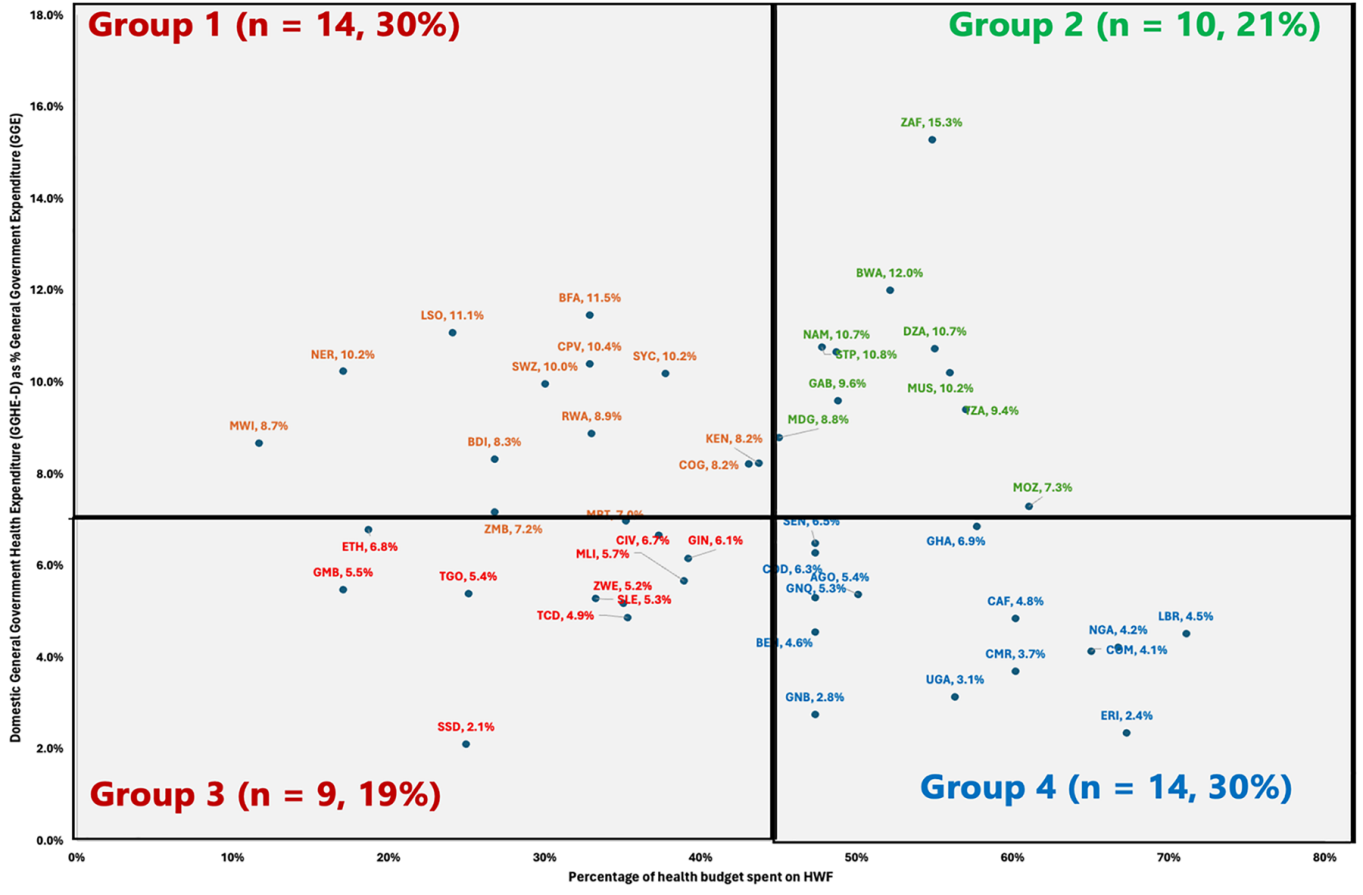
Prioritisation for Health and Health Workforce in the WHO African Region for 2022.

Comparing the level of prioritisation for health from general government spending and the level of HWF prioritisation from the health spending revealed that in 49% of the countries (see Figure 2), there is room to improve HWF investments by better prioritising HWF within the existing health expenditure envelope. Of these, 19% (n = 9) can also improve the prioritisation of health spending from the overall government expenditure envelope. Also, as shown in Figure 2, for 51% of the countries, improving HWF investments could principally stem from exploring efficiency gains, while 30% of countries (n = 14) could benefit from advocating for improving the prioritisation of health spending from the overall government expenditure envelope. To illustrate, Figure 2 groups the countries into 4.

• **Group 1 countries:** These countries have higher than the regional average level of prioritisation of health within Government spending (>7%) but less than the regional average level of prioritisation of HWF within the health spending (<43%). Almost 1 in 3 countries (30%, n = 14) are in this group, where the quick wins for expanding HWF investments could come from better prioritisation of HWF within existing health budgets. As appropriate, advocacy should be used to increase the overall health budget or get external aid.
• **Group 2 countries:** These countries have higher than the regional average level (>7%) of prioritisation of health within Government spending and also higher than the regional average level of prioritisation of HWF within the health spending (>43%). Ten countries (21% of the Region) are in this so-called frontier group, where the quick wins for expanding HWF investments could stem mainly from allocative efficiency gain from existing HWF spending, then advocacy to increase the overall health budget or get external aid, as appropriate.
• **Group 3 countries:** These countries have lower than the regional average level (<7%) of prioritisation of health within Government spending and also lower than the regional average level of prioritisation of HWF within the health spending (<43%). About 1 in 4 countries (n = 9, 19% of the Region) are in this group, where HWF investment is highly constrained. These are countries that need maximum political commitment to increase prioritisation of health within government spending but, most importantly, increase the proportion of health spending allocated to the health workforce.
• **Group 4 countries:** These countries have lower than the regional average level of prioritisation of health within Government spending (<7%) but higher than the regional average level of prioritisation of HWF within the health spending (>43%). Almost a third of countries are in this group (n = 14, 30%), where room to increase HWF investments comes mainly from increasing the level of prioritisation of health investments from the overall government spending or mobilising external development assistance for the health workforce.

### Estimates of the budget space for health workforce employment

This study modelled 3 scenarios of health workforce investment (expenditure): (1) business as usual scenario, (2) HWF prioritisation scenario and (3) health prioritisation scenario.

In the ‘business as usual’ scenario, the public sector budget space, private sector contribution and support from overseas development assistance together suggested a potential budget envelope of US$20.85 billion in 2022, which, if all the macroeconomic parameters considered in the analysis remain relatively constant, could increase by quarter (26%) to US$26.34 billion in 2026 and a further 21% to US$31.81 billion by 2030. However, volatility in the volume and flows of overseas development assistance heightens uncertainties of the ballpark estimate. Under the same ‘business as usual’ scenario, if only the public sector envelope is considered, the potential funding was estimated to be US$14.17 billion in 2022, which would improve by 21% to US$17.24 billion by 2026 and could reach US$20.67 billion by 2030. Table 3 provides detailed estimates for each across the 3 scenarios considered in the analysis.

**Table 3.** Ballpark estimates of potential budget envelopes for the health workforce.

Country	Public sector (business as usual scenario)			Public sector + Private sector + ODA (business as usual scenario)			Public sector + Private sector + ODA (Enhanced HWF prioritisation scenario)			Public sector + Private sector + ODA (Overall health prioritisation scenario)		
	2022	2026	2030	2022	2026	2030	2022	2026	2030	2022	2026	2030
Algeria	1744.01	2282.74	2250.44	2447.51	3203.56	3158.23	3421.25	4478.09	4414.72	3421.25	4478.09	4414.72
Angola	268.51	220.41	268.77	376.82	309.31	377.19	1053.69	864.92	1054.72	1053.69	864.92	1054.72
Benin	37.79	55.46	73.07	53.03	77.83	102.54	174.65	256.32	337.71	174.65	256.32	337.71
Botswana	352.12	438.23	568.56	383.30	477.04	618.90	478.89	596.00	773.24	478.89	596.00	773.24
Burkina Faso	144.48	204.62	264.07	221.40	313.56	404.66	221.51	313.72	404.88	289.68	410.28	529.48
Burundi	27.01	26.10	37.99	56.66	54.77	79.69	63.65	61.52	89.52	102.24	98.82	143.80
Cabo Verde	14.76	20.45	25.87	24.89	34.49	43.64	27.46	38.05	48.14	35.93	49.79	62.98
Cameroon	114.55	155.65	191.13	160.75	218.43	268.23	651.83	885.72	1087.65	651.83	885.72	1087.65
Central African Republic	5.99	8.31	10.29	8.40	11.66	14.44	26.02	36.11	44.72	26.02	36.11	44.72
Chad	8.23	9.82	11.64	16.89	20.16	23.90	42.70	50.96	60.43	51.99	62.06	73.59
Comoros	3.19	4.23	4.57	5.52	7.31	7.91	20.06	26.58	28.76	20.06	26.58	28.76
Côte d’Ivoire	181.85	265.10	347.36	231.34	337.25	441.90	451.99	658.92	863.39	521.07	759.61	995.32
Democratic Republic of the Congo	192.85	262.55	375.15	270.64	368.46	526.48	647.47	881.48	1259.53	647.47	881.48	1259.53
Equatorial Guinea	69.69	61.72	67.24	97.80	86.61	94.36	277.19	245.49	267.46	277.19	245.49	267.46
Eritrea	2.47	2.65	2.84	3.47	3.73	3.98	22.11	23.74	25.36	22.11	23.74	25.36
Eswatini	27.94	31.24	35.46	56.49	63.18	71.70	59.30	66.33	75.27	85.00	95.07	107.89
Ethiopia	1079.10	2094.42	2760.07	2737.33	5312.87	7001.43	2623.63	5092.21	6710.63	6045.12	11,732.97	15,461.98
Gabon	103.53	103.66	117.06	128.11	128.26	144.85	200.34	200.59	226.52	200.34	200.59	226.52
Ghana	286.90	324.81	414.30	291.29	329.79	420.65	637.72	722.00	920.92	637.72	722.00	920.92
Guinea	81.21	117.32	153.09	155.83	225.15	293.78	346.57	500.73	653.37	380.17	549.27	716.71
Guinea-Bissau	3.69	5.50	7.16	5.18	7.72	10.05	28.25	42.08	54.75	28.25	42.08	54.75
Kenya	501.98	574.54	719.11	763.12	873.44	1093.21	1390.46	1591.46	1991.89	1390.46	1591.46	1991.89
Lesotho	23.91	26.85	30.52	59.94	67.32	76.51	45.45	51.04	58.01	81.19	91.17	103.63
Liberia	9.35	12.55	16.64	12.81	17.20	22.79	42.52	57.09	75.65	42.52	57.09	75.65
Madagascar	89.55	116.81	159.26	125.67	163.93	223.50	214.46	279.75	381.41	214.46	279.75	381.41
Malawi	21.68	20.39	25.17	100.45	94.49	116.63	47.16	44.37	54.76	173.83	163.52	201.84
Mali	70.63	96.44	118.82	112.94	154.21	190.01	270.34	369.15	454.84	298.84	408.06	502.78
Mauritania	39.25	47.95	55.60	67.92	82.97	96.21	119.56	146.06	169.37	146.03	178.39	206.86
Mauritius	89.89	109.82	118.61	126.16	154.12	166.45	185.48	226.59	244.73	185.48	226.59	244.73
Mozambique	162.04	229.91	373.72	227.40	322.65	524.48	467.90	663.88	1079.17	467.90	663.88	1079.17
Namibia	151.42	182.82	213.21	175.01	211.29	246.42	246.44	297.53	347.00	246.44	297.53	347.00
Niger	46.60	69.66	91.88	149.56	223.55	294.86	86.95	129.98	171.44	219.28	327.76	432.32
Nigeria	688.37	755.67	1209.69	690.69	758.22	1213.77	2452.44	2692.21	4309.72	2452.44	2692.21	4309.72
Republic of Congo	76.14	93.57	115.34	106.85	131.31	161.86	195.06	239.72	295.48	195.06	239.72	295.48
Rwanda	15.11	17.36	23.05	121.58	139.71	185.45	35.81	41.15	54.62	205.31	235.91	313.15
São Tomé and Príncipe	5.27	8.72	11.86	7.40	12.23	16.65	10.31	17.05	23.20	10.31	17.05	23.20
Senegal	131.46	201.95	265.86	207.89	319.36	420.43	481.14	739.14	973.06	481.14	739.14	973.06
Seychelles	20.24	24.90	31.96	32.39	39.86	51.15	41.85	51.50	66.08	47.73	58.74	75.37
Sierra Leone	5.13	5.11	7.00	12.23	12.17	16.69	26.82	26.69	36.58	34.70	34.54	47.33
South Africa	6647.54	7097.00	7965.90	9035.37	9646.27	10 827.28	9035.37	9646.27	10 827.28	9035.37	9646.27	10 827.28
South Sudan	3.74	3.47	4.42	10.95	10.14	12.92	45.24	41.90	53.41	77.96	72.20	92.03
Tanzania	343.05	449.92	649.36	481.43	631.41	911.31	768.04	1007.32	1453.85	768.04	1007.32	1453.85
The Gambia	1.91	2.73	3.42	6.75	9.65	12.09	7.33	10.48	13.13	18.47	26.41	33.09
Togo	14.32	20.13	26.12	41.24	57.97	75.20	67.06	94.27	122.29	114.94	161.57	209.59
Uganda	83.46	120.19	166.09	146.52	211.02	291.60	700.21	1008.44	1393.51	700.21	1008.44	1393.51
Zambia	97.54	118.80	159.18	157.72	192.11	257.39	205.60	250.43	335.52	330.21	402.21	538.88
Zimbabwe	85.04	136.32	126.98	134.27	215.24	200.50	316.92	508.02	473.22	389.35	624.14	581.38
Overall	14 174.44	17 238.57	20 674.87	20 846.90	26 342.98	31 813.86	28 982.23	36 273.04	44 860.92	33 478.35	44 268.07	55 322.04

In the ‘HWF prioritisation’ scenario where all parameters are constant, but countries prioritise at least 43% of their health budget for HWF employment, the overall HWF envelope could increase by 28% from the ‘business as usual’ scenario (as shown in Table 3). In 2022, the ‘HWF prioritisation’ scenario would have resulted in a combined regional HWF envelope of US$28.98 billion. It could increase to US$36.27 billion by 2026 and US$44.86 billion in 2030 if the macroeconomic parameters (GDP, inflation, unemployment, and trade balance) remain favourable.

A ‘Health Prioritisation’ Scenario assumed countries prioritised at least 15% of public sector consumption (general government spending) for health – in line with the Abuja target – but maintained prevailing levels of HWF prioritisation from the health budget. This scenario would have increased the HWF envelope by at least 60% in 2022 from the ‘business as usual’ scenario and could yield US$44.27 billion in 2026 and potentially US$55.32 billion by 2030.

### Funding gaps to absorb the stock of health workers in 2022

In 2022, about 4.17 million health workers required US$36.3 billion for wage bill, including the employment of those that were unemployed compared with the estimated envelope from all sources of US$20.85 billion, leaving a rough financing deficit of 43% in 2022 if all trained health workers were to be employed – whether in the public sector, private sector or through overseas development assistance (Table 4). As highlighted in Box 1, without the contribution of the private sector and overseas development assistance, the funding gap to maintain existing wage bill and employ the unemployed health worker is about 61%.

**Table 4.** Investments required to maintain the existing wage bill, absorb trained health workers and scale up training and employment.

Occupation	Stock of health workers in 2022*	Median Annual Salary (USD)	Investment to employ all the stock health workers in 2022 (and maintain current jobs) – in Million USD
Medical doctors	369 145	16 711.45	6168.95
Nursing personnel	1 698 828	7534.15	12 799.22
Midwifery personnel	334 530	8216.37	2748.62
Pharmacist	101 401	11 129.03	1128.50
Dentist	34 404	24 675.15	848.94
Other health workers	1 628 411	7741.29	12 606.00
Total	4 166 720		36 300.22

* Occupations in the stock exclude 7 non-core health occupations (with a reported headcount of 931 557, 18% of the entire workforce).

Box 1. **Investment gaps to absorb currently trained and unemployed health workers.**

• The public sector envelope for the health sector alone covers approximately 39% of the resources required to employ the current stock of health workers, **leaving a budgetary gap of 61%.**

• If the existing volume and mix of HWF spending from the private sector and overseas development assistance are considered, the anticipated budget envelope could cover **57% of the wage bill of the existing stock of health workers, still leaving a gap of almost 43%.**

## Discussion of Findings and Limitations

Building on our previous work^6^ and recent conceptual discourse on budget space analysis for health^14,15^ while using a simple model, we modelled ballpark estimates of the potential expenditure envelope for health workforce employment for all 47 countries in the WHO African region under 3 scenarios. In a ‘business as usual’ scenario, we assumed that government spending on health remained constant while macroeconomic factors like GDP growth drove the budget space. The estimated budget increased from US$20.85 billion in 2022 to US$31.81 billion by 2030. Volatility in overseas development assistance affects the estimate. Prioritising 43% of the health budget for the health workforce (HWF), employment increased the HWF envelope by 28%. Prioritising 15% of public sector consumption for health could yield US$55.32 billion by 2030.

With the current levels of funding for the health workforce, the WHO African Region is unlikely to halve the current health worker shortage as up to a US$15.45 billion investment gap is required to absorb all unemployed health workers, representing 43% of current spending level, taking into account government, private sector spending as well overseas development assistance. This financing gap translates into unemployed health workers, estimated to be 27% (95% CI: 14%-39%) based on data from a subset of 10 countries conducting health labour market analyses. This level of budget-induced paradoxical unemployment is widespread, and several countries face a shortage of health workers at the frontlines of service delivery, but almost 1 in 3 cannot find suitable jobs.^10,11,13,30^ As the Region is estimated to face a 6.1 million shortage of health workers, more significant investments are required, estimated to be US$120 billion by 2030.^25^ Despite the seemingly large amount of funds required for the health workforce, the required investment is less than 2% of the GDP of countries in the African Region. African countries spend roughly 5% of GDP on health, of which 29% is spent on workforce employment.^26^

The analysis also revealed one-size-fits-all approach would not be appropriate for countries as they are at different levels of prioritisation of health and health workforce investments. For example, in 49% of the countries, there appears to be room to improve health workforce investments if the health workforce is better prioritised within the existing health expenditure envelope. The need to reprioritise health workforce investments as a share of total health expenditure should, meanwhile, be multi-pronged, including getting top-level political commitment to secure investment in health, including the health workforce on an unprecedented scale, as happened during the COVID-19 pandemic,^31^ improving efficiency in health workforce spending.^32^

By this, health workforce advocates could draw the parallels between increased investments in the health workforce, health outcomes and economic growth and use the growing evidence for increased advocacy. While, globally, it has been established that the higher the density of the health workforce, the better the levels of health outcomes, including life expectancy,^33^ an empirical investigation into this commutative relationship revealed that increasing life expectancy by just a year boosted workers’ productivity, and increased economic growth by 3.66% and foreign direct investment inflows by 1.62 units in Ghana.^34,35^

There are several limitations that should be considered when interpreting the findings of this study. Firstly, this study did not account for health workforce related (in)efficiency due to data limitations, the impact efficiency on budget space is well documented.^36-39^ Despite African health systems being said to becoming more efficient,^40^ about 20% of all health investments are reportedly lost to technical inefficiencies, of which the health workforce is known to be a contributor.^36,40-43^ As identified in the current study, 14 countries (30% of the Region) showed higher-than-average levels of prioritisation of health and health workforce in the overall government spending, implying that exploring efficiency gains is the starting point for expanding the expenditure envelope in those countries. Nonetheless, nearly all countries can improve efficiency in the health workforce by improving the quality of budgetary and financial management,^44^ improving equitable distribution and reducing absenteeism of health workers, which has lately become a significant source of health workforce inefficiencies.^45,46^ However, these efficiency-enhancing health workforce reforms are not cost-neutral in themselves and have been shown to take 3 to 10 years to realise their benefits.^47^

Secondly, data availability and quality presented a challenge. In 14 countries, there was no publicly available data on private sector contribution to spending on health workforce remuneration, overseas development assistance contribution to HWF remuneration. Also, in 8 countries, there was no publicly available data on the proportion of health spending allocated to the health workforce. In these instances, the regional average was used to input for the missing data. Additionally, in some countries, the latest available data predates the COVID-19 pandemic, during which important health budget decisions were made.

Thirdly, although this study used direct government spending measures, the findings must be interpreted cautiously as most data is subject to change based on future economic conditions. Thus, these estimates should be considered as order of magnitude (or ballpark) estimates intended to provide a rough idea of the potential envelope for advocacy purposes and unsuitable for operational planning in countries.

Finally, best practice guidelines recommends that modelling studies include sensitivity analysis on the input parameters and structural assumptions. However, in this study, we relied on secondary data for the input parameters which had no estimates of the boundaries of uncertainty. As a result, we could not conduct sensitivity analysis of the input parameters. We, therefore, resorted to varying the assumptions in the form of scenarios which are reported in the paper as part of the results. This should, however, be considered a weakness and future efforts should explore ways of overcoming it.

## Conclusion and Policy Options

Our estimates suggest that better prioritising health within the overall government expenditure coupled with highly prioritising health workforce to at least regional average levels of 43% within the health budget could make a difference in the investments needed in tackling the health workforce challenges. This should, however, be underpinned by ensuring efficiency and accountable use of the resources. In line with the principles of the Africa Health Workforce Investment Charter,^48^ several policy and strategic options could be pursued to expand investments in the employment of health workers while ensuring efficiency.

First, engage multisectoral policy dialogue to initiate policy reforms through investment compacts to foster alignment between donors and the government in prioritising health workforce needs. This alignment should be based on the national HWF strategy and the national health sector strategic plan. Investments can be made more efficiently and streamlined by ensuring that HRH funding is in line with the country’s strategic priorities.

Second, establish wage harmonisation by aligning wages for specific cadres of health workers, both domestically and those supported by external sources, according to evidence-informed target wage levels. This can be achieved through a salary harmonisation approach, which promotes equitable compensation across different health worker categories.

Third, advocate for on-budget external support for health workforce financing to enhance transparency and visibility of donor-supported workforce financing. This support should be aligned with government priorities and adhere to approved wages and remuneration for health worker cadres within the government establishment. This approach enables the transition of externally financed personnel onto domestic funding as donor resources shrink. It also allows for discontinuing off-budget and ad hoc/off-establishment HWF support that may create distortions. Fourth, regular and comprehensive analyses of the wage bill should be conducted to identify inefficiencies, including head count duplications, ghost workers and other payroll irregularities. By addressing these issues, the health workforce can be optimised, and efficiency gains made in the health workforce wage bill can be used to finance new recruitments.

Finally, health workforce analytics can be strengthened by implementing health labour market analyses, establishing health workforce accounts, and deploying agile human resource information systems. These systems should provide real-time data on health workforce financing and facilitate periodic payroll cleaning. Additionally, they should enable the right-sizing of the workforce to align with service delivery needs. By enhancing data-driven decision-making, countries can better manage their health workforce and respond to changing demands.

## Supplemental Material

sj-xlsx-1-his-10.1177_11786329251320429 – Supplemental material for Ballpark Estimates of Budget Space for Health Workforce Investments in the 47 Countries of the WHO African Region: A Modelling Study Supplemental material, sj-xlsx-1-his-10.1177_11786329251320429 for Ballpark Estimates of Budget Space for Health Workforce Investments in the 47 Countries of the WHO African Region: A Modelling Study by James Avoka Asamani, San Boris Kouadjo Bediakon, Hamza Ismaila, Sunny Okoroafor, Regina Titi-Ofei, Adam Ahmat, Juliet Nabyonga-Orem, Ogochukwu Chukwujekwu and Kasonde Mwinga in Health Services Insights
